# Tumor microbiome metabolism: A game changer in cancer development and therapy

**DOI:** 10.3389/fonc.2022.933407

**Published:** 2022-07-25

**Authors:** Xiaozhuang Zhou, Shruthi Kandalai, Farzana Hossain, Qingfei Zheng

**Affiliations:** ^1^ Department of Radiation Oncology, College of Medicine, The Ohio State University, Columbus, OH, United States; ^2^ Center for Cancer Metabolism, James Comprehensive Cancer Center, The Ohio State University, Columbus, OH, United States

**Keywords:** tumor microbiome, metabolism, cancer therapy, cancer development, immune response

## Abstract

Accumulating recent evidence indicates that the human microbiome plays essential roles in pathophysiological states, including cancer. The tumor microbiome, an emerging concept that has not yet been clearly defined, has been proven to influence both cancer development and therapy through complex mechanisms. Small molecule metabolites produced by the tumor microbiome through unique biosynthetic pathways can easily diffuse into tissues and penetrate cell membranes through transporters or free diffusion, thus remodeling the signaling pathways of cancer and immune cells by interacting with biomacromolecules. Targeting tumor microbiome metabolism could offer a novel perspective for not only understanding cancer progression but also developing new strategies for the treatment of multiple cancer types. Here, we summarize recent advances regarding the role the tumor microbiome plays as a game changer in cancer biology. Specifically, the metabolites produced by the tumor microbiome and their potential effects on the cancer development therapy are discussed to understand the importance of the microbial metabolism in the tumor microenvironment. Finally, new anticancer therapeutic strategies that target tumor microbiome metabolism are reviewed and proposed to provide new insights in clinical applications.

## Introduction

The human microbiota is a broad category consisting of diverse bacteria, fungi, protists, archaea, and viruses that occur in and on the human body (1). The total number of these microbes is believed to be more than 100 trillion, which amounts to 2 kg in mass (2). Due to its important pathophysiological role in human health and disease, the microbiome has also been referred to as “the last human organ under active research” (3) and “the second brain” (4). Moreover, the number of unique genes from the microbiome is estimated to be 100-fold higher than that from human cells, as noted by the NIH Human Microbiome Project (5, 6). The proteins encoded by these genes and the metabolites biosynthesized by these microbes are able to influence not only their own microbial communities, but also the biological functions of host cells (7, 8). Notably, small molecule metabolites secreted by the human microbiome affect local and systemic bodily functions, including energy generation, metabolism of dietary components, biosynthesis of vitamins, immune responses, behavior, and even mood (9–11).

While microbes were implicated in diseases long ago, the contributions of the tumor microbiome to carcinogenesis, cancer progression, metastasis, and treatment have been poorly understood until recently (12–14). Previous studies have shown that microbes belonging to the genera *Salmonella* and *Helicobacter* affect cellular dysplasia and carcinogenesis (15, 16). Microbiota homeostasis can also play a role in cancer development (17). For instance, dysbiosis is associated with the carcinogenesis of gastrointestinal (GI) and non-GI tumors while also acting as an oncogenic driver of colorectal cancer (CRC) (18). Current research indicates that human-associated microbes interact with host cells and affect disease states, especially cancer, *via* diverse mechanisms (19, 20). One key mechanism is microbial metabolites serving as small molecule messengers to mediate crosstalk between microbes and host cells (21). Specifically, microbial metabolites can alter the tumor microenvironment (TME) (22), which includes inflammatory mediators, recruited immune cells, fibroblasts, adipocytes, endothelial cells, and pericytes (22, 23), thereby directly influencing cancer progression (23, 24) and the efficacy of immunotherapy (1, 23). One well-studied example of this is the genotoxic metabolite colibactin, produced by pathogenic *Escherichia coli*, that can directly induce DNA double-strand breaks (DSBs) (25), thus motivating CRC development (26).

As the tumor microbiome metabolism exhibits direct and indirect impacts on cancer development, novel therapy strategies may be developed by targeting these unique metabolic pathways (27, 28). Chemical biology, synthetic biology, and biomedical engineering approaches facilitate the remodeling of the microbiome-containing TME and will provide new opportunities for the future development of bacterial, viral, chemical, and immunological therapies.

In this review, we intend to highlight the tumor microbiome and how it affects cancer development and therapy as a new game changer. Among the multiple crosstalk mechanisms between microbes and cancer cells, we specifically focus on the unique metabolites produced by the tumor microbiome. The chemical structures and biochemical mechanisms through which tumor microbiome metabolism affects cancer biology are addressed. Finally, yet importantly, the potential clinical applications of targeting tumor microbiome metabolism through multidisciplinary methods for future cancer therapy have been proposed and discussed.

## What is tumor microbiome?

**Figure 1:**
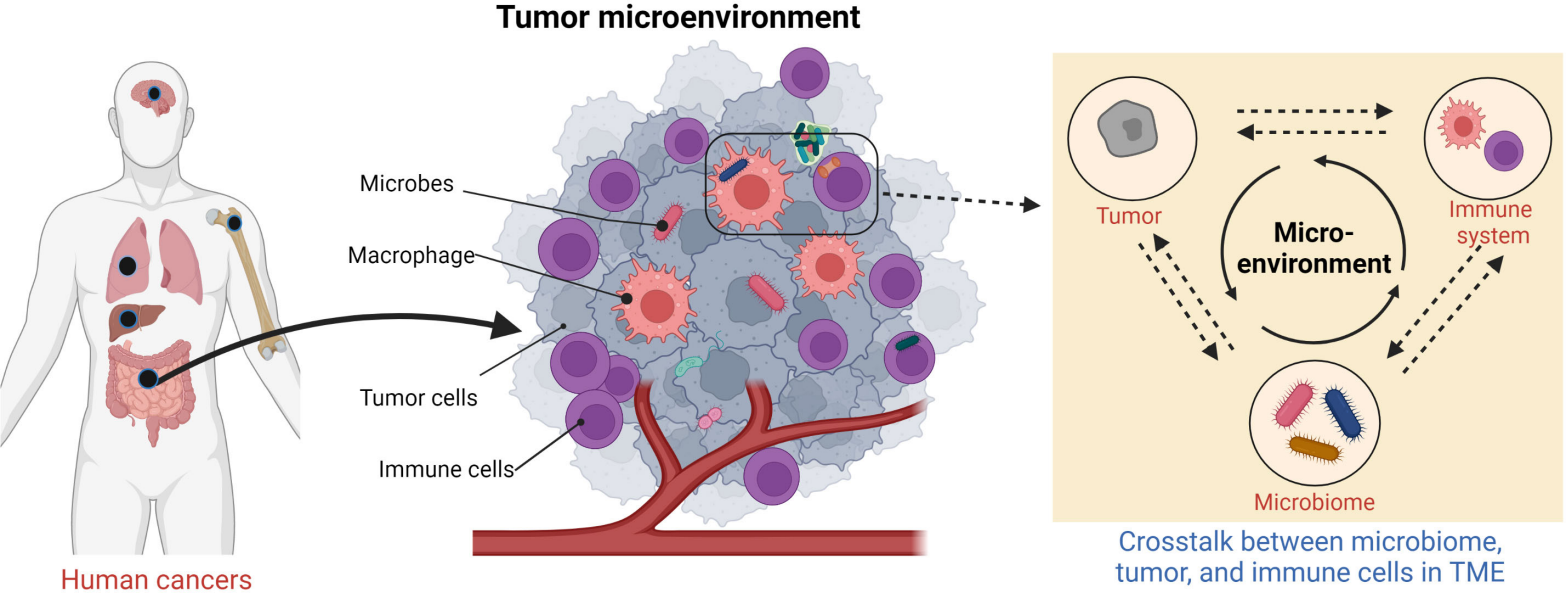
Schematic of human tumor microenvironment that contains tumor microbiome.

The tumor microbiome is an emerging concept that has yet to be clearly defined. It broadly refers to all microorganisms located within the TME (**Figure 1**) and encompasses bacteria, fungi, archaea, viruses, and other microbes (29) that contribute to the reshaping of the microenvironment. These microbes are widespread in the TME and inhabit inside or outside the tumor cells and immune cells. It has long been in debate whether these microbes constitute a predetermined niche or rather represent a transient stochastic colonization (29).

Within cancer biology, intratumoral bacteria and their effects are a newly raised concept (30). While bacteria were observed in tumor isolates previously, it was assumed that these were contaminants and were not associated with cancer cells (31). Recently, a large-scale analysis of over 1,500 clinical samples indicated that the majority of the tumor microbiome is intracellular bacteria that exhibit tumor-site-specific properties (32). Intratumoral bacteria and host cancer cells mutually influence each other through the transcriptome and metabolome (33). Since these intracellular bacteria inhabit cancer cells, direct crosstalk between host and microbes is easily mediated by biomacromolecules and small molecule metabolites. However, this still leads to a chicken-and-egg situation—is the accumulation of intratumoral bacteria a cause or effect of cancer? Further investigations are required to address this question. Intracellular microbes hiding inside other type of cells, such as macrophages and fibroblasts, have also been shown to remodel the TME (34, 35) and thus affect cancer development and treatment (36, 37).

On the other hand, viruses that directly cause cancer (also known as oncoviruses) have been thoroughly studied. These viruses currently include hepatitis B virus (HBV), hepatitis C virus (HCV), human papillomaviruses (HPVs), Kaposi’s sarcoma-associated herpesvirus (KSHV/HHV-8), human T-lymphotropic virus (HTLV), Merkel cell polyomavirus (MCV), and Epstein–Barr virus (EBV) (38). They induce cancer through diverse mechanisms, such as the integration of viral DNA into the host genome (39) and the inactivation of tumor suppressor genes like p53 and Rb (40). Globally, these oncoviruses are associated with approximately 10%–16% of cancer cases (41, 42). It has also been suggested that other viruses, similar to the bacteria mentioned previously, may play a role in carcinogenesis, without directly causing cancer (37). Other microbes, such as fungi, have also been implicated in cancer (43, 44), although this is less studied.

Extracellular microorganisms in the TME, such as those in the gut microbiota, oral microbiota, vaginal flora, and skin flora, also play essential roles in cancer development (45–47) and have significant impacts on curative outcomes (48). For instance, it has long been known that the colonization by *Helicobacter pylori* in stomach can directly cause gastric cancer (49), as well as gastric mucosa–associated lymphoid tissue (MALT) lymphoma (50). As a result, *H. pylori* is associated with approximately 5% of cancers worldwide (42). Multiple studies have shown that the gut microbiota interacts with the host by producing of a diverse set of metabolites and toxins from exogenous dietary substrates and endogenous host cellular compounds (51). Host metabolic disorders are systematically associated with alterations in the composition and function of the gut microbiota (52). Specific classes of microbiota-derived metabolites, notably bile acids (BAs), short-chain fatty acids (SCFAs), branched-chain amino acids, trimethylamine N-oxide, and tryptophan and indole derivatives, have been implicated in the pathogenesis of host cell metabolic disorders, some of which directly relate to carcinogenesis (53). In addition, the gut microbiome is essential in shaping the development of innate and adaptive immunity (54) and plays an essential role in the clinical efficiency of cancer immunotherapy (55).

## Crosstalk between tumor microbiome and cancer cells

**Figure 2:**
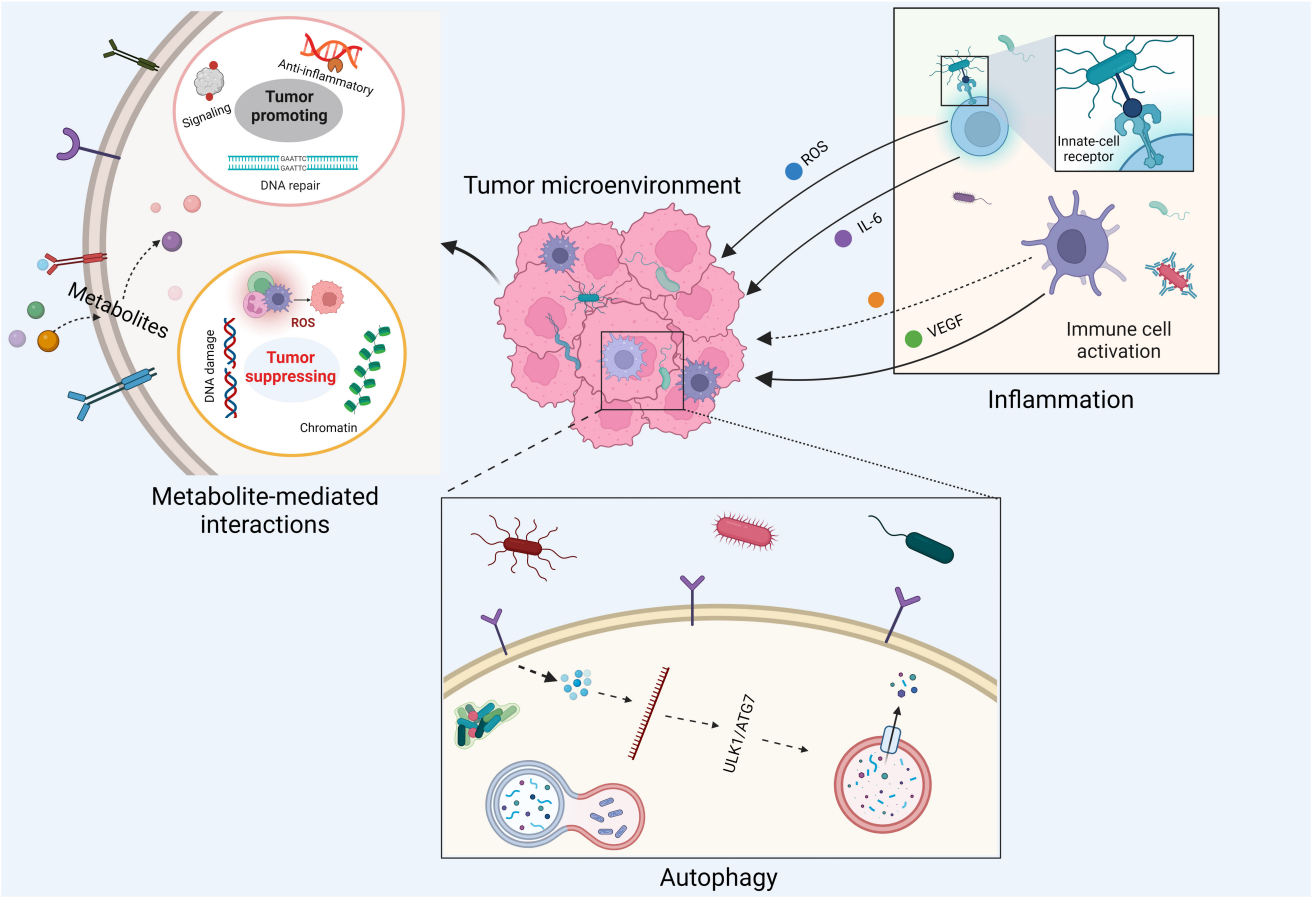
Impacts of tumor microbiome on cancer development.

The crosstalk between the tumor microbiome and cancer cells is diverse and complex, involving cell–cell direct interactions and messenger molecule-mediated effects (**Figure 2**). With respect to host cell–microbe direct interactions, intracellular microbe–induced autophagy and extracellular microbe–caused inflammation are two well-studied examples. For instance, it has been shown that *Fusobacterium nucleatum* modulates the autophagy pathways of CRC cells by targeting TLR4 and MYD88 innate immune signaling and specific microRNAs, thereby promoting CRC chemoresistance and migration (56). Moreover, it has been accepted for decades that inflammation is a critical component of tumor progression (57). Inflammatory cells significantly influence the TME, thereby affecting neoplastic processes and fostering the proliferation, survival, and migration of cancer cells (58). Chronic, dysregulated, persistent, and unresolved inflammation is associated with an increased risk of malignancies, as well as the malignant progression of most types of cancer (58). As microorganisms are one of the major causes of inflammation, the tumor microbiome can manipulate cancer development by remodeling the TME through the recruitment of inflammatory cells. In fact, it has been pointed out that bacterial infections can trigger chronic inflammation that leads to host cell proliferation and tumor development (59).

Messenger molecule–mediated interactions between host cells and microbes are another key machinery linking the tumor microbiome to cancer progression. These messenger molecules involve secreted proteins, peptide toxins, and small-molecule metabolites. For example, the virulence factor cytolethal-distending toxin produced by *Campylobacter jejuni* is one of the major causes for infectious diarrhea worldwide and has been shown to induce carcinogenesis *in vivo* (60, 61). Moreover, tumor microbiome–derived small molecule metabolites can reach remote tumor entities through systemic circulation, free diffusion, and active transport (such as the transport of lactate and pyruvate by proton-coupled monocarboxylate transporters) (62). These metabolites are able to stimulate antitumoral or carcinogenic innate immune responses (22) *via* non-covalent interactions. For instance, evolutionary conserved pathogen-associated molecular patterns (PAMPs) from commensal microbes or pathogens can be systematically sensed by the innate immune system *via* pattern recognition receptors, such as Toll-like receptors and NOD-like receptors, leading to the host’s innate immune responses (63). There is evidence showing that bacterial PAMPs can boost antitumor immunity by augmenting Toll-like receptor signaling and serving as cancer vaccine adjuvants (64–66). Additionally, commensal gut bacteria can recruit natural killer T immune cells to control the growth of liver tumors *via* their unique microbial metabolism of BAs (67). Moreover, chemically reactive metabolites from the tumor microbiome can promote or inhibit tumor growth through the covalent modifications of DNA, RNA, histones, and other essential enzymes involved in host signaling transduction pathways (68). These modifications can be enzymatic or non-enzymatic and are capable of inducing cancer-causing and cancer-promoting epigenetic changes of host cells (69). As a result of this complex crosstalk between the host and tumor microbiome, both cancer and immune cells change their own metabolic status to adapt to the reshaped TME (70).

Furthermore, due to its novel metabolic and catabolic pathways, the gut microbiome is capable of converting human-ingested nutrients into functional microbial metabolites that closely link diet, cancer, and other metabolic diseases (19, 71, 72). These microbial metabolites produced by microbes from diet, such as BAs and SCFAs, have significant impacts on cancer and immune cells (73–78), thereby affecting cancer development and immunotherapies through complex mechanisms (79–81). Based on the important role of the microbiome in connecting diet and different types of cancer, recent research advances have suggested that gut microbiota modulation would become a novel strategy for prevention and treatment of CRC (82). As diet and microbial communities affect one another, dietary interventions have proven to be an efficient approach to modulate the intestinal microbiota, which is in line with the growing recognition of significant impacts of diet and lifestyle on human health through microbiome regulation (83).

## Metabolites produced by tumor microbiome

The consequence of metabolism is the production of small molecule metabolites, which are typically classified into two categories: primary metabolites and secondary metabolites. Primary metabolites are compounds that are directly involved in an organism’s growth and development, while secondary metabolites are not directly involved in these processes and tend to vary more by species (84). There are a number of primary metabolites produced by microbes that contribute to cancer development or suppression, such as methylglyoxal (MGO), SCFAs, BAs, reactive oxygen species (ROS), amines, and methane (CH_4_) (85–87). These molecules are biosynthesized by diverse human-associated microorganisms, including archaea (88), bacteria (89, 90), fungi (90) protists (91) and parasites (91, 92).

**Figure 3:**
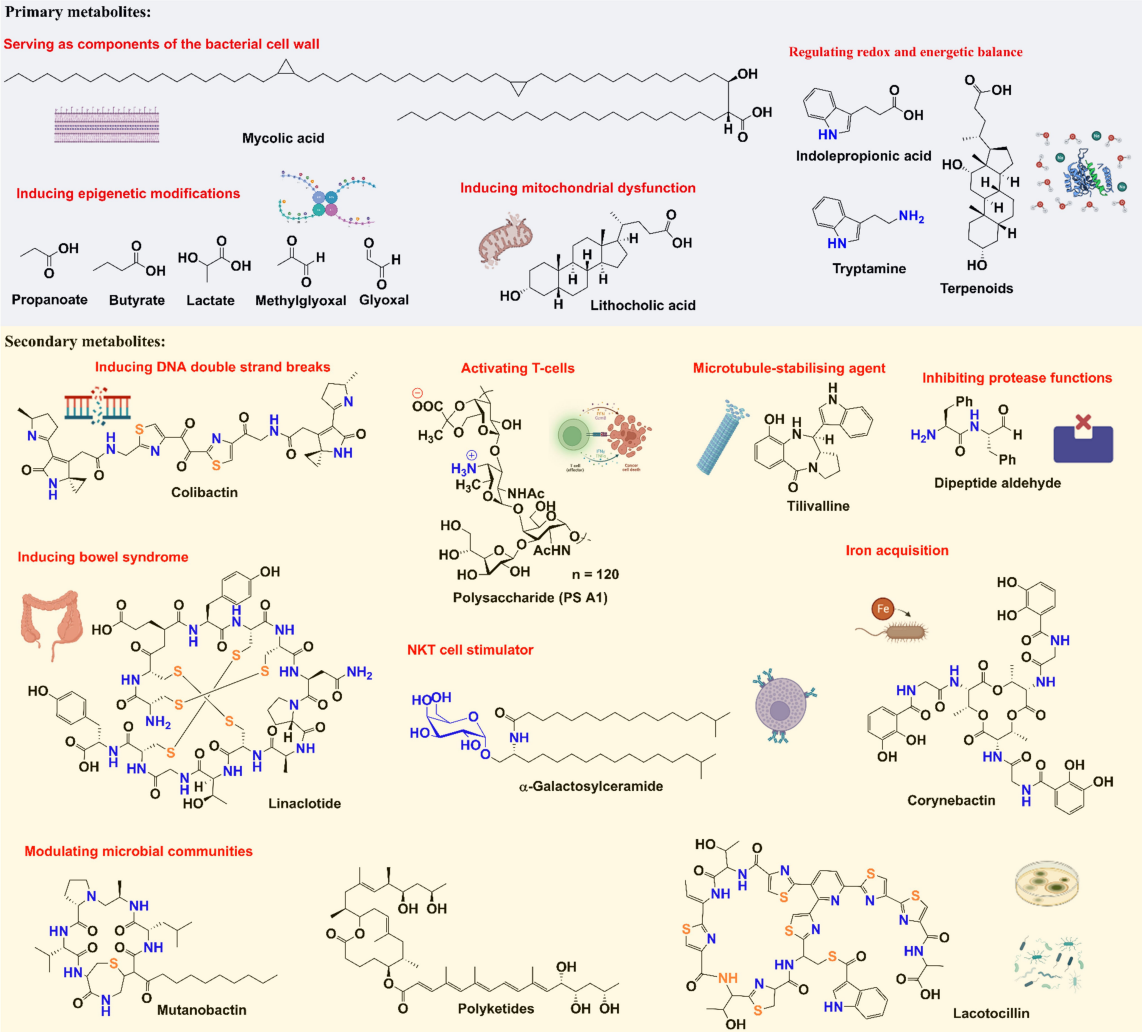
Chemical structures and functions of representative metabolites from tumor microbiome.

There are several examples of secondary metabolites with well-established functions, such as colibactin, peptide aldehyde, and thiopeptide, that have been known to affect cancer development, and these metabolites have diverse chemical structures (**Figure 3**). As a well-studied secondary metabolite molecule, colibactin is a cytotoxin mainly produced by pathogenic *Escherichia coli*, as well as other members of the family *Enterobacteriaceae*. The production of colibactin was shown to have a direct and significant association with CRC *via* the induction of DNA DSBs (25, 26). Peptide aldehydes were discovered as metabolites from a variety of microbes (including *E. coli*, *Bacillus subtilis*, and *Streptomyces* species) and are known to inhibit protease functions (93, 94), which may increase carcinogenicity. Thiopeptides have complex structures and strong antibacterial activities (95, 96), which can affect the distribution of human flora (97). In addition to being isolated from multiple environmental microbes, thiopeptides have been discovered from many microbial species in various parts of the body, including *Lactobacillus gasseri* in the urogenital tract, *Propionibacterium acnes* on the skin, *Streptococcus downei* in the oral cavity, and *Enterococcus faecalis* in the gut (98). Moreover, emerging studies have suggested that thiopeptides may also serve as anticancer agents by targeting proteasomes and transcription factor FOXM1 (99).

## Impacts of tumor microbiome metabolites on cancer development

Since small molecule metabolites from tumor microbiome play essential roles in cancer development, we would like to summarize some examples in this section to emphasize the neglected but significant impacts of tumor microbiome metabolism on the TME (**Figure 3**). As stated above, colibactin’s ability to cause DNA DSBs allows it to promote tumorigenesis (100). Recently, it has been shown that colibactin also targets bacteria by triggering prophage induction (101), which may explain how this metabolite further affects the communities in the tumor microbiome.

SCFAs are mainly bacterial fermentation products from starch and other polysaccharides (102) and include a wide range of molecules including acetate, propionate, butyrate, and lactate (89). Among these, butyrate has been shown to potently inhibit the activity of histone deacetylases (103–105), whereas propionate does so moderately and acetate has no effect (106, 107). Lactate is known to play significant roles in the Warburg effect and reverse Warburg effect (108–110), as well as affect chromatin biology through histone modification (111, 112). It has also been shown that SCFAs can: 1) modulate macrophage functions by promoting the production of nitric oxide, IL-6, IL-12 (113), and IL-22 (114); 2) induce the differentiation of T_reg_ cells (115–117); and 3) regulate the migration of neutrophils (118). There are many connections between SCFAs and cancer, where SCFAs function as a double-edged sword in tumorigenesis. SCFAs have been implicated to have cancer-promoting or cancer-suppressing effects that vary under different conditions and with different types of cancer. Previous research has shown that SCFAs are able to: inhibit human colon cancer invasion (119, 120), inhibit the migration and invasion of fibrosarcoma cells (121), increase IGF1 levels to promote the proliferation of prostate cancer cells (122), upregulate proapoptotic protein BAK (123), downregulate adhesion protein α_2_β_1_ integrin (124), induce cell stress responses and apoptosis in colorectal cells (125), inhibit proliferation and increase differentiation and apoptosis of adenocarcinoma cells (126), impair hypoxia-induced angiogenesis (127), and regulate p53 expression (128, 129).

BAs are steroid derivatives that play essential regulatory roles in the GI system and cancer development. While primary BAs are produced by the liver, secondary BAs, mainly deoxycholic acid and lithocholic acid, occur when primary BAs are further metabolized by gut bacteria. Secondary BAs have long been proposed to promote tumors (130). In addition, further derivatives of secondary bile salts can cause apoptosis, increase ROS production, and lessen pro-apoptotic effects (131). Deoxycholic acid is believed to be associated with oncogenic mutations of proto-oncogene *KRAS* (132) and can lead to DNA DSBs and apoptosis (133). Lithocholic acid has been shown to modulate T_h_17 and T_reg_ cells (73), inhibit HLA class I genes (134), and induce endoplasmic reticulum stress and mitochondrial dysfunction in human prostate cancer cells (135). Moreover, CRC cells can obtain resistance to apoptosis after being exposed to specific bile salts (136, 137).

Polyamines are small molecule metabolites with two or more amino groups, which exhibit a variety of functions. The most common polyamines, putrescine, cadaverine, spermidine, and spermine, are metabolized from arginine (138) but can also be produced by gut bacteria (139, 140). Polyamines are known to protect cells from ROS (141) due to their reducing activities and have been significantly correlated with CRC (142, 143). Polyamines have been shown to be associated with inhibiting the growth of prostate cancer cells (144–146), downregulating estrogen receptor α in breast cancer cells (147), serving as a downstream effector from *H. pylori*, leading to DNA damage and immune cell apoptosis in stomach cancer (148–151), and increasing the risk for development of skin cancer in mouse models (152, 153). Moreover, microbial polyamines exhibit unique activities in the regulation of macrophage polarization and function, thereby affecting host immune responses (154).

MGO is a chemically reactive dicarbonyl metabolite of glucose metabolism (155, 156). In mammalian cells, MGO is mainly generated as a byproduct through a non-enzymatic dephosphorylation process during glycolysis, although it can also be produced by tumor microbes that contain microorganism-specific methylglyoxal synthases (88, 157). MGO can react with nucleophilic groups of biomacromolecules, such as lysine and arginine residues in proteins (158), as well as guanine residues in DNA and RNA (159). This MGO-induced non-enzymatic covalent modification (glycation) can result in the formation of advanced glycation end products (AGEs) (160–162) and changes in the three-dimensional chromatin architecture (163–165). It has been shown that elevated levels of MGO in the TME lead to the overexpression of an MGO detoxifier, glyoxalase I (Glo1), in cancer cells (166, 167). There is evidence showing that low concentrations of MGO are beneficial for cancer cell growth, while high levels of MGO contribute negatively to cell survival by disrupting multiple signaling pathways (168, 169). The biphasic model proposed recently is a convincing explanation for the function of MGO-induced glycation in manipulating chromatin damage and cancer cell survival (166). Moreover, the recently identified histone MGO-glycation eraser and rewriter enzymes, DJ-1 and PAD4, have been recognized to possess cancer-promoting effects as oncoproteins (163, 164). Thus, developing deglycase activity–oriented high-throughput screening assays for identifying DJ-1 and PAD4 inhibitors will provide new insights for the mechanistic studies of host deglycation pathways, as well as clinical applications (170).

## Targeting tumor microbiome for cancer therapy

**Figure 4:**
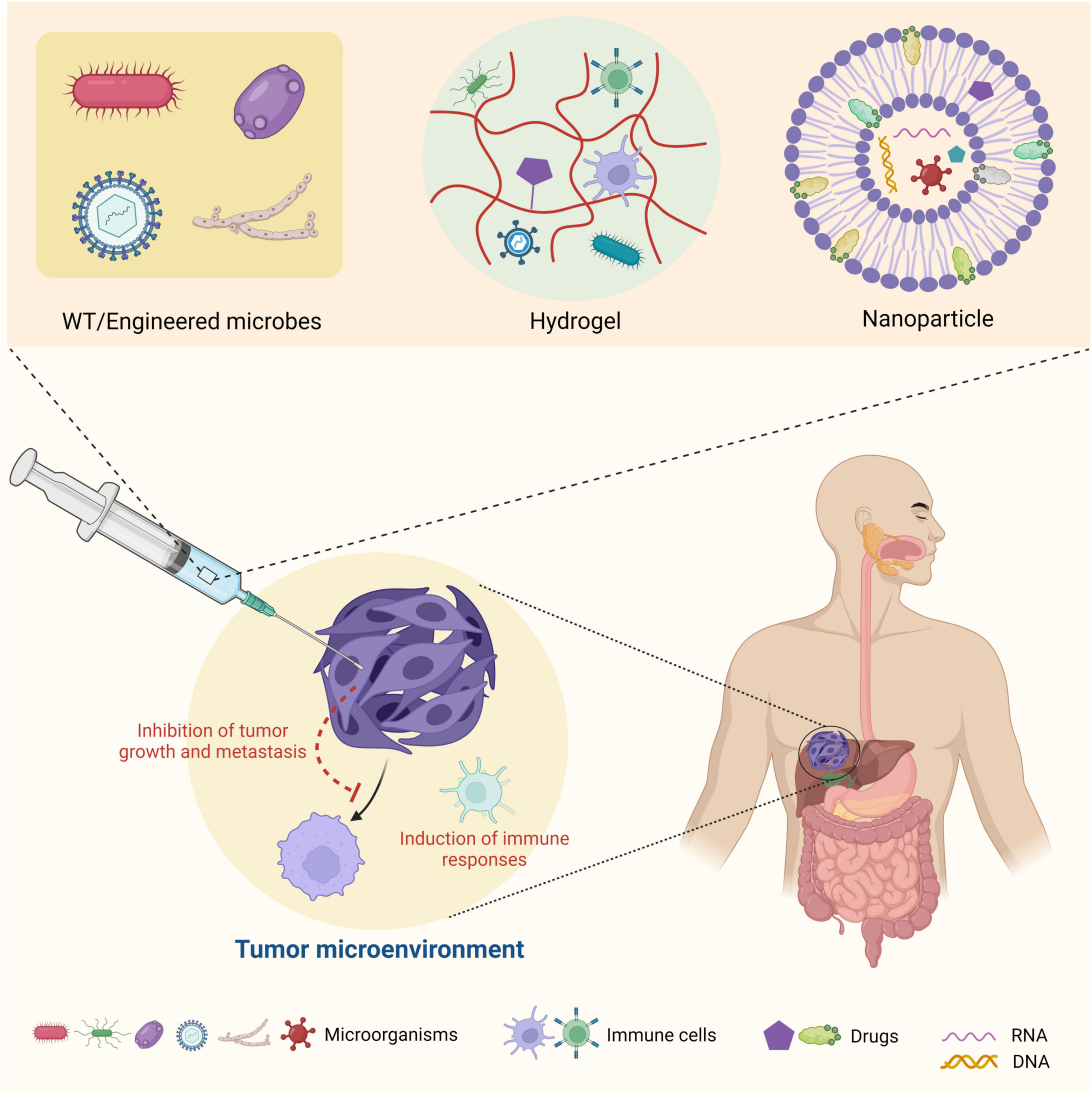
Summary of therapeutic strategies targeting tumor microbiome metabolism.

As noted above, due to the inseparable connections between microbes, host immune cells, and cancer cells, targeting the tumor microbiome seems to be a practical tactic for cancer therapy (**Figure 4**). Specifically, strategies include the development of wild-type and/or engineered microbes for bacterial and viral therapies and the application of chemical biology, synthetic biology, and biomedical engineering to target the tumor microbiome metabolism for reshaping TME. Ideally, with a deeper understanding of the tumor microbiome’s function in the TME and cancer development, we could build up an artificial ecosystem of microorganisms in the TME to prevent cancer cells from spreading and enhance the efficiency of immunotherapy.

Based on their functions in suppressing or promoting cancer progression, microbes within the TME can be classified to “good bugs” or “bad bugs” for cancer therapies (171). A straightforward treatment strategy is to take advantage of “good bugs” and get rid of “bad bugs” in the TME. For example, *Enterococcus* species have been noted to promote responses to immune checkpoint immunotherapy (ICI) (172). *Bifidobacterium pseudolongum* and *Akkermansia muciniphila* were observed to produce the metabolite inosine, which enhances ICI through T_h_1 activation (173). Following biomaterial modulation, mice with increased levels of *Peptostreptococcus anaerobius* and reduced levels of other bacterial species responded better to oral squamous cell carcinoma ICI (174). Bacteria belonging to the *Gammaproteobacteria* family have been found to inactivate the chemotherapy drug gemcitabine, which is often used for the treatment of pancreatic ductal adenocarcinoma (175). Overall, modulating the microbial communities in the TME can provide new opportunities for cancer therapies (176). Accordingly, synthetic biology approaches have been applied to engineer specific tumor microbiome species to develop enhanced bacteria-based cancer therapies. For instance, as low concentrations of L-arginine can cause poor responses to PD-L1 ICI, probiotic strain *E. coli* Nissle 1917 was engineered to convert ammonia to L-arginine, thereby increasing T-cell infiltration and enhancing ICI (177). Additionally, Nissle 1917 and other *E. coli* strains were engineered to release nanobodies with diverse functions to motivate T-cell infiltration and tumor shrinkage (178, 179). There are also a number of clinical trials in various phases regarding the applications of engineered bacteria for cancer therapies, some of which have shown promising results (180) (**Table 1**).

**Table 1:** Representative microorganisms applied for cancer therapy.

Microorganism	Clinical Phase	Cancer Type	Status (Trial Identifier)
***Salmonella* Typhimurium VNP20009**	I	Metastatic melanoma or renal cell carcinoma	Results published (N/A)
***Salmonella* Typhimurium TAPET-CD (VNP20009 expressing cytosine deaminase)**	I	Head and neck solid cell carcinoma or esophageal adenocarcinoma	Results published (N/A)
***Salmonella* Typhimurium (χ4550 expressing human IL-2)**	I	Liver metastases of solid tumors	Results published (NCT01099631)
***Salmonella* Typhimurium VXM01 (Ty21a expressing VEGFR2)**	I	Pancreatic cancer	Completed (NCT01486329)
***Clostridium novyi-*NT**	I	Solid tumor malignancies	Results published (NCT01924689)
***Clostridium novyi-*NT**	Ib	Treatment-refractory advanced solid tumors	Recruiting (NCT03435952)
**CRS-100 (live-attenuated *Listeria monocytogenes*)**	I	Liver metastases of solid tumors	Completed (NCT00327652)
***Listeria monocytogenes***	II	Metastatic pancreatic tumors	Results published (NCT01417000)
***Listeria monocytogenes***	II	Cervical cancer	Results published (NCT01266460)
**VE800 (11 commensal bacteria strains)**	I/II	Metastatic cancer, melanoma, gastric cancer, or colorectal cancer	Active (NCT04208958)
***MET-4* bacterial strains**	N/A	Locoregionally-advanced oropharyngeal squamous cell carcinoma	Recruiting (NCT03838601)
***Enterococcus* strain MNC-168**	I	Advanced malignant solid tumors	Not yet recruiting (NCT05383703)
***Lactobacillus johnsonii* LA1 and *Bifidobacterium longum* BB536**	II	Colorectal cancer	Completed (NCT00936572)
***Plasmodium vivax***	I/II	Non-small cell lung cancer	Unknown (NCT02786589)
***Plasmodium vivax***	I/II	Advanced breast cancer or advanced liver cancer	Unknown (NCT03474822)
***Agaricus bisporus* extract**	I	Breast cancer recurrence	Completed (NCT00709020)
***Agaricus bisporus* extract**	I	Prostate cancer recurrence	Completed (NCT00779168)
***Trametes versicolor* extract**	I	Breast cancer	Completed (NCT00680667)
***Ganoderma lucidum* spore**	II	Non-small cell lung cancer	Unknown (NCT02844114)
***Ganoderma lucidum***	III	Pediatric cancers	Completed (NCT00575926)
**Modified measles virus**	I	Mesothelioma	Completed (NCT01503177)
**Modified measles virus**	I	Ovarian cancer and peritoneal cavity cancer	Results published (NCT00408590)
**GL-ONC1 (modified vaccinia virus)**	I	Solid tumors	Completed (NCT00794131)
**M032 (modified herpes simplex virus)**	I	Glioblastoma, astrocytoma, or gliosarcoma	Active (NCT02062827)
**G207 (modified herpes simplex virus)**	I/II	Glioblastoma, astrocytoma, or gliosarcoma	Completed (NCT00028158)
**H101 (modified adenovirus)**	N/A	Gynecological cancer	Recruiting (NCT05051696)
**Modified fowlpox virus and modified vaccina virus**	II	Prostate cancer	Completed (NCT00003871)
**Talimogene laherparepvec (modified herpes simplex virus)**	III	Melanoma	Results published (NCT00769704)
**Pexastimogene Devacirepvec (modified vaccinia virus)**	III	Hepatocellular carcinoma	Results published (NCT02562755)

Microorganisms including bacteria (in blue), protists (in orange), fungi (in green), and viruses (in gray) have been utilized in clinical trials for cancer treatment. All information is from ClinicalTrials.gov.

Similarly, oncolytic virotherapy has also been applied as an immunotherapy for cancer treatment (181–183). For example, alphavirus M1 was identified for such use, as it specifically targets cancer cells deficient in zinc-finger antiviral protein (184). Engineered oncolytic viruses expressing PD-L1 inhibitors have clinical potentials for curing cancers resistant to PD-1/PD-L1 ICI, as they are able to activate tumor neoantigen–specific T-cell responses (185). Notably, virotherapy has been approved in some countries for use against cancer. Imlygic, which is engineered from herpes simplex virus I (HSV1) and contains granulocyte-macrophage colony-stimulating factor, was approved in 2015 by the US Food and Drug Administration and European Medical Agency for the treatment of melanoma (186). G47Δ, which is engineered from HSV1, was approved in 2021 by Japan Ministry of Health, Labor and Welfare for the treatment of malignant glioma and other brain cancers (187). Oncorine, which is engineered from adenovirus, was approved in 2005 by the China Food and Drug Administration Department in combination with chemotherapy for the treatment of nasopharyngeal carcinoma (186). Moreover, there are other oncolytic virotherapies engineered from HSV1, adenovirus, and measles virus currently in various phases of clinical trials (186) (**Table 1**).

The toxins and chemicals extracted from microbes can also be used for cancer treatment. This strategy dates back to the late 19th century when Coley’s toxins (a mixture of toxins filtered from killed *Streptococcus pyogenes* and *Serratia marcescens*) were utilized to cure cancer (188). Although this was an unstable approach with poor repeatability, the application of Coley’s toxins led to milestone breakthroughs in immuno-oncology, such as the discovery of tumor necrosis factor α (TNF-α) (189). TNF-α has since been identified to suppress tumor growth and improve the efficacy of immunotherapy by activating cell death pathways (190, 191). Commensal bacteria have been found to play significant roles in CpG-oligodeoxynucleotide immunotherapy, which depend on the increased production of TNF-α (192). Microbial SCFAs have also been shown to improve CAR-T cell therapy by enhancing the levels of TNF-α in different cancer models (193).

Last but not least, recent advances in biomedical engineering have provided new opportunities for cancer treatment by targeting the tumor microbiome. For example, the utilization of biomaterials, such as nanoparticles (194, 195) and hydrogels (174), to modulate and deliver microbial communities to specific sites of the TME opens a new door for future cancer therapies (**Figure 4**). These novel materials can be designed to be stimuli responsive (196) and utilized for the controlled and targeted release of toxic chemotherapy drugs (197), therapeutic antibodies (198, 199), CAR-T cells (200, 201), or live microbes to reshape the TME (202–204). These applications of new biomaterials will offer a promising platform for basic and translational research and will accelerate clinical outcomes of drugs that may have poor solubility and high toxicity.

## Outlook and perspectives

In this review, we have summarized the research process of the tumor microbiome, mainly focusing on the impacts of its unique microbial metabolism on cancer development and therapy. Over the past few decades, microorganisms have been regarded only as a cause of infectious disease. The pathophysiological functions of human-associated microbes have long been neglected until recently when the microbiome was identified to manipulate and affect diverse disease states, as well as therapeutic efficacy. The impacts of the human microbiome are so broad that research papers on the topic have exploded in the past few years. Accordingly, a number of new concepts have been raised to describe the omnipotent human microbiota, including the “brain-gut axis” and “second brain.” Despite these, the tumor microbiome still lacks a precise definition. Nevertheless, the tumor microbiome plays constructive roles in cancer biology, some of which are still elusive. Among these macro- and micropathophysiological effects induced by the tumor microbiome, small molecule metabolite–mediated crosstalk appears to be particularly important due to the free diffusion of metabolites that can easily impact local and distant tumor tissues *via* covalent modifications and/or non-covalent interactions. Here, we have provided representative examples to emphasize the role of tumor microbiome metabolism as a game changer in cancer biology and clinical treatment, as well as its broad biomedical effects that were once disregarded.

Targeting the pathways of microbial metabolism and crosstalk between host and microbes will provide future avenues for cancer diagnosis, treatment, and recovery. Accordingly, therapy strategies have been developed at distinct levels to target tumor microbiome metabolism: 1) directly applying wild-type or engineered live microbes in immuno-oncology; 2) utilizing the microbial-extracted fractions or synthetic chemicals that interfere with corresponding metabolic pathways for cancer treatment; and 3) utilizing rationally designed biomaterials to rebuild a benign TME by modulating the microbial ecosystem. All in all, after having a deeper understanding of the close correlation between the tumor microbiome and human cancer, we would change our perception of these microorganisms’ identities in tumor tissues from “short-term tenants” to “permanent residents.”

## Author contributions

QZ proposed the conception, wrote, and edited the manuscript. XZ drafted the manuscript and figures. SK participated drafting and editing the manuscript as well as references. FH drafted and edited the chemical structures. All authors listed in the paper have made a substantial, direct, and intellectual contribution to the work and approved it for publication.

## Funding

This study is supported by OSUCCC startup funds for QZ.

## Conflict of interest

The authors declare that the research was conducted in the absence of any commercial or financial relationships that could be construed as a potential conflict of interest.

## Publisher’s note

All claims expressed in this article are solely those of the authors and do not necessarily represent those of their affiliated organizations, or those of the publisher, the editors and the reviewers. Any product that may be evaluated in this article, or claim that may be made by its manufacturer, is not guaranteed or endorsed by the publisher.
